# An Extragastrointestinal Tumor Diagnosed as a Vaginal Mass during Pregnancy

**DOI:** 10.1155/2022/7879220

**Published:** 2022-10-25

**Authors:** Reo Ando, Shuichi Taniguchi, Shuji Arita, Murasaki Aman, Kousuke Marutsuka, Tomihiro Shimamoto

**Affiliations:** ^1^Department of Obstetrics and Gynecology, Miyazaki Prefectural Miyazaki Hospital, Miyazaki, Japan; ^2^Department of Chemotherapy, Miyazaki Prefectural Miyazaki Hospital, Miyazaki, Japan; ^3^Department of Pathology, Miyazaki Prefectural Miyazaki Hospital, Miyazaki, Japan

## Abstract

We report a case of an extragastrointestinal stromal tumor diagnosed as a vaginal mass during pregnancy. The mass was detected during routine examination at 24 weeks of gestation. At 26 weeks, the patient underwent transvaginal ultrasonography and magnetic resonance imaging, which revealed a blood flow-rich mass of approximately 50 × 30 mm in the rectovaginal septum. At 29 weeks of gestation, we resected the mass vaginally and the pathological diagnosis was a gastrointestinal stromal tumor. Chemotherapy was withheld until after full-term birth because the proliferation index of the tumor cells was low. The patient delivered a healthy infant. Imatinib was commenced at 1 month postpartum, with no recurrence or metastasis after 2.5 years. An extragastrointestinal stromal tumor as a vaginal mass in pregnancy has not been reported; however, our case suggests that the tumor should be considered a differential diagnosis of a vaginal mass in pregnancy.

## 1. Introduction

Gastrointestinal stromal tumors (GISTs) are mesenchymal tumors that usually arise from the wall of the gastrointestinal tract. GISTs outside the gastrointestinal tract are called extraGISTs (EGISTs). EGISTs account for a small percentage of GISTs and are commonly located in the mesentery [1]. The epidemiology of vaginal EGISTs is unknown, but the reported number of cases of vaginal leiomyoma is approximately 300 since 1733, suggesting that vaginal GISTs are very rare. There are few reports on EGISTs related to obstetrics and gynecology, with 17 cases in the vagina and five cases in the rectovaginal septum [2, 3]. Thirteen cases during pregnancy have been reported, but all cases developed in locations other than the vagina [4]. To the best of our knowledge, cases of EGISTs in the vagina during pregnancy have not been reported. We report a case of an EGIST as a vaginal mass in a 38-year-old pregnant woman.

## 2. Case Presentation

**Figure 1:**
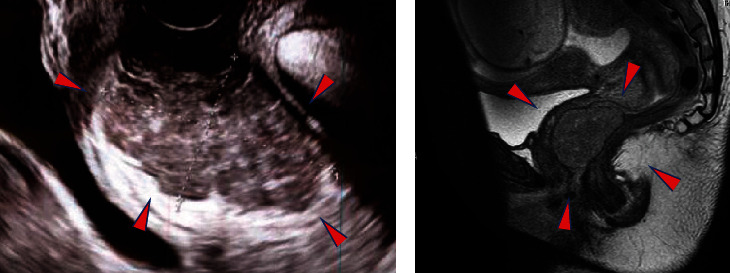
Radiographic images showing the mass. (a) Transvaginal ultrasound: the mass (arrowheads) was 51.7 × 32.5 mm in size. (b) Plain magnetic resonance image of the abdomen and pelvis. An irregular mass (arrowheads) can be seen superior to the vagina.

A 38-year-old Japanese woman, gravida 3 para 2, presented to our hospital with a vaginal mass at 29 weeks of gestation. The pregnancy was diagnosed at a maternity clinic at 6 weeks of gestation. At 24 weeks, during a routine pregnancy medical examination, a vaginal mass was incidentally found. She had no significant medical history. Her grandmother had gastric cancer, and her grandfather had lung cancer. She was referred to our hospital for a detailed investigation of the mass and for treatment. Prior to the pregnancy, she felt some discomfort, and although a mass was present in her perineum, its origin could not be ascertained. Pelvic examination revealed an elliptical, nontender mass, approximately 5 cm in size in the posterior vaginal wall. The mass projected into the vagina and was easily detectable and mobile. Rectal examination revealed an intact rectal mucosa. Transvaginal ultrasound revealed a 51.7 × 32.5 mm mass with hypervascularity (Figure 1(a)). Magnetic resonance imaging (MRI) of the pelvis showed a tumor measuring 52 × 37 × 35 mm, which was isointense compared to muscle on T1-weighted imaging and heterogeneously hyperintense on T2-weighted image. The boundaries of the mass were clear and did not appear to involve the rectum and vagina. The mass was in the rectovaginal septum, but its origin was uncertain (Figure 1(b)). No evidence of metastasis was observed.

We consulted the surgeons about the treatment policy. We diagnosed the tumor as benign and resected it per vagina at 29 weeks of gestation, considering that the mass could potentially cause bleeding and obstruction during delivery. Under general anesthesia, with the patient in the lithotomy position, the mass was carefully resected via the vagina to avoid rupture; the surgical margins were not wide enough to make the operation minimally invasive.

**Figure 2:**
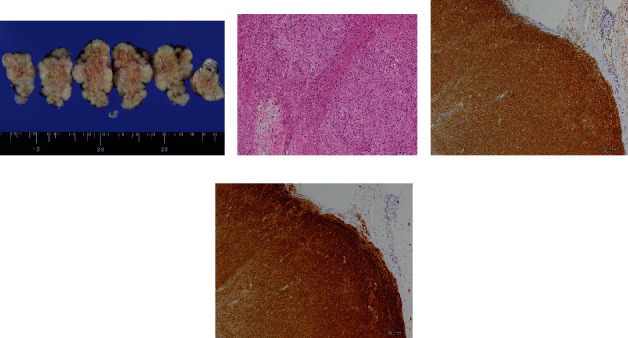
Pathological findings. (a) Macroscopic findings: the cut surface of tumor was smooth, light brown, and solid, and no necrosis was observed. (b) On hematoxylin and eosin staining: spindle-shaped cells with nuclear atypia were arranged in a bundle and had proliferated. (c, d) On immunohistochemistry examination: CD34 and CD117 (KIT) were positive.

The resected mass measured 53 × 44 mm, was irregular in shape, and had a smooth edge. On the cut section, the tumor was light brown, and there was no evidence of rupture (Figure 2(a)). On hematoxylin and eosin staining, there were well-demarcated, spindle-shaped cells with oval to elongated nuclei, proliferating in fascicular and streaming patterns. Mitosis and apoptosis were occasionally seen; typical mitoses were 2–4/50 high-power fields (Figure 2(b)). Immunohistochemically, the tumor cells were positive for CD34 (Figure 2(c)) and CD117 (KIT) (Figure 2(d)) but negative for SMA, desmin, and S100 protein. These findings were consistent with GISTs. The Ki-67-labeling index was 3.6%. The pathological diagnosis was GIST. According to the modified Fletcher classification, we noted the tumor as high risk for recurrence based on the tumor size and the primary site [5]. In addition, genetic analysis revealed a deletion-insertion mutation of exon 11 of the c-kit gene at codons 557–559 (p. Trp557-Val559delinsPhe). No other mutations were detected. This mutation type is a poor prognostic factor in general GIST [6, 7]. In this case, adjuvant imatinib was recommended [8]. However, the effects of imatinib on the fetus are not well known. A low mitotic index and Ki-67-labeling index indicated that the tumor was not aggressive at the time. These proliferation indicators and tumor size are the most powerful prognostic factors [9]. Therefore, we commenced imatinib treatment postpartum.

The postoperative and antenatal courses were favorable. She was followed up with transvaginal ultrasound and pelvic examination weekly until delivery. She delivered vaginally at 40 weeks of gestation. The baby weighed 3754 g, which was appropriate for gestational age. The APGAR scores at the 1^st^ and 5^th^ minutes after birth were 9 and 9, respectively. She was administered adjuvant imatinib 400 mg/day one month after delivery without breastfeeding to avoid the effects of the drug on the baby. There were no significant adverse reactions after drug administration and no recurrence at 2.5 years after delivery. We intend to continue this therapy for 3 years and maintain a long-term follow-up.

## 3. Discussion

The most distinctive feature of this case was that the EGIST was found as a vaginal mass. GISTs originating from the vaginal wall or rectovaginal septum are rare. According to the report by Cheng et al., five cases of GIST/EGIST were located in the vagina and 15 cases in the rectovaginal septum. In their report, the average age of the patients was 54 years, and the clinical manifestations of GIST originating from the rectovaginal cavity were nonspecific symptoms, including massive bleeding, pain, abdominal distension, pollakiuria, and constipation, all of which had nonspecific compression effects. These symptoms seemed related to the location of the mass. The findings of GIST in computed tomography (CT) and MRI are nonspecific, and cases are diagnosed based on pathological findings [2]. In another report, an EGIST presenting as a vaginal mass caused symptoms such as copious vaginal bleeding, which required emergency medical care, with associated spontaneous discharge of tumoral tissue per vaginam [10]. Such symptoms were not found in our case, but could have been obscured by the pregnancy. We considered the differential diagnosis to be vaginal fibroids, vaginal cancer, hematoma, etc. and performed various detailed examinations before surgery, but no findings suggestive of malignancy were obtained. As mentioned by Cheng et al. [2], it is difficult to arrive at the correct diagnosis preoperatively in the case of EGIST cases.

GISTs are rarely found during pregnancy. Ten cases of patients diagnosed with GISTs during pregnancy were reported by Tanaka et al. [3, 4], excluding recurrent cases and a case diagnosed in the puerperium. We have provided the details of these cases in the Supporting Information. The median age of the 10 patients was 31 years, and the median number of gestational weeks at the time of consultation was 19.5 weeks. Abdominal pain was the most common reason for consultation in three cases, which is the same as that for general GIST. However, there were three cases of incidental detection during medical examination for pregnancy, which is common for GIST during pregnancy. Ultrasonography and MRI were the main preoperative examinations, and CT was performed in one case. Biopsy was performed in 3 cases, and GIST was diagnosed in 2 cases. One of the effects of GIST on labor and the fetus in a study was anemia due to bleeding from the tumor. The origin of the GIST was gastric in four cases and extragastrointestinal in two cases, including the omentum and retroperitoneum.

Adjuvant imatinib administered for 3 years to patients with a high risk of GIST recurrence after macroscopic complete resection surgery was associated with prolonged recurrence-free survival and overall survival. The unstratified Cox proportional hazards model (HRs) for overall survival observed suggests that a 3-year treatment may prevent approximately 50% of deaths during the first decade of follow-up compared with 1 year of imatinib [11]. However, the effect of imatinib on a baby during breastfeeding is not well known. As per the European Leukemia Net Recommendations, imatinib may be secreted in breast milk and breastfeeding is not recommended [12]. These data suggest the importance of a definitive diagnosis of GIST and appropriate treatment. An EGIST presenting as a gynecological mass is very rare, but EGISTs should be considered in the differential diagnosis of vaginal masses during pregnancy.

## Data Availability

The data that support the findings of this study are openly available in PubMed.

## Supplementary Materials

Supplementary Materials Table: characteristics of gastrointestinal stromal tumors (GISTs) during pregnancy.
