# Supplementary material for: An Extragastrointestinal Tumor Diagnosed as a Vaginal Mass during Pregnancy
Source: Case Rep Obstet Gynecol. 2022 Oct 25;2022:7879220. doi: 10.1155/2022/7879220 (PMC9626234; doi:10.1155/2022/7879220)
Supplement: Supplementary Materials — Table: characteristics of gastrointestinal stromal tumors (GISTs) during pregnancy. [file 7879220.f1.docx]

**Supporting information**

Table. Characteristics of gastrointestinal stromal tumors (GISTs) during pregnancy

|  | Reference | Maternal age | Gestational week at diagnosis | Reason for consultation | Preoperative diagnosis | Origin |
| --- | --- | --- | --- | --- | --- | --- |
| 1 | Stubbs^1^ | 31 | 16th | Abdominal mass | GIST | Transverse mesocolon |
| 2 | Lanzafame^2^ | 29 | 22nd | Abdominal pain | Stomach mass | Stomach |
| 3 | Valente^3^ | 32 | 28th | Abdominal pain | - | Stomach |
| 4 | Charif^4^ | 42 | 20th | Biliary colic pain | Cystic mass | Stomach |
| 5 | Tanaka^5^ | 29 | 14th | Bloody vomiting | Stomach tumor | Stomach |
| 6 | Haloob^6^ | 31 | 19th | Shortness of breath | GIST | Small intestine |
| 7 | Scherjon^7^ | 25 | 10th | Uterus enlargement | Ovarian mass | Intestine |
| 8 | Igras^8^ | 42 | 20th | Routine ultrasound | Retroperitoneal mass | Duodenum |
| 9 | Gozukara^9^ | 21 | 15th | Abdominal pain | Leiomyoma uteri | Omentum |
| 10 | Coveney^10^ | 42 | 23rd | Ultrasound scan | Lymphoma or renal mass | Retroperitoneal |
| 11 | This case | 38 | 29th | Vaginal mass | Benign vaginal mass | Rectovaginal septum or Vagina |

**Supporting information references**

1. Stubbs BM, Desai A, Singh S, Seddon B, Khan F. Gastrointestinal stromal tumour in pregnancy. *BMJ Case Rep* 2011. doi: 10.1136/bcr.01.2011.3737
2. S. Lanzafame, V. Minutolo, R. Caltabiano, et al. About a case of GIST occurring during pregnancy with immunohistochemical expression of epidermal growth factor re- ceptor and progesterone receptor. *Pathol Res Pract* 2006; 202: 119-123.
3. Valente PT, Fine BA, Parra C, Schroeder B. Gastric stromal tumor with peritoneal nodules in pregnancy: tumor spread or rare variant of diffuse leiomyomatosis. *Gynecol* Oncol 1996; 63: 392-397.
4. Charif I, Khalil N, Ousadden A, et al. Pregnancy with gastric stromal tumor. *Case Rep* *Clin* 2014; 03: 571-576.
5. Tanaka N, Tamada S, Ueno N, Ishida M, Kodama J, Kubota T. A rare maternal gastrointestinal stromal tumor found in the second trimester of pregnancy: A case report. *Case Rep Womens Health* 2020; 28: e00251.
6. Haloob N, Slesser AA, Haloob AR, Khan F, Bostanci G, Abdulla A. An elective combined caesarean section and small bowel GIST resection during the third trimester of pregnancy: report of a case. *Int J Surg Case Rep* 2013; 4: 121-124.
7. Scherjon S, Lam WF, Gelderblom H, Jansen FW. Gastrointestinal stromal tumor in pregnancy: a case report. *Case Rep Med* 2009. [doi.org/10.1155/2009/456402](https://doi.org/10.1155/2009/456402)
8. Igras ET, Fosh BG, Neuhaus SJ. Maternal GIST in twin pregnancy: case report of a rare and complex management challenge. *Gynecol Oncol Case Rep* 2012; 2: 133-135.
9. Gözükara I, Dilek TUK, Durukan H, Apa DD, Kucur SK, Dilek S. Extragastrointestinal stromal tumor during pregnancy. *Case Rep Obstet Gynecol* 2012. [doi.org/10.1155/2012/846747](https://doi.org/10.1155/2012/846747)
10. Coveney SD. Twin pregnancy complicated by an adnexal mass. *Australas J Ultra- sound Med* 2011; 14: 31-33.
